# The Inhibitory Potential of 2′-dihalo Ribonucleotides against HCV: Molecular Docking, Molecular Simulations, MM-BPSA, and DFT Studies

**DOI:** 10.3390/molecules27144530

**Published:** 2022-07-15

**Authors:** Ahmed Khalil, Amany S. El-Khouly, Eslam B. Elkaeed, Ibrahim H. Eissa

**Affiliations:** 1 Department of Chemistry, College of Science, King Faisal University, Al Ahsa 31982, Saudi Arabia; aalkholy@kfu.edu.sa; 2 Chemistry Department, Faculty of Science, Zagazig University, Zagazig 44519, Egypt; 3 Department of Chemistry, Faculty of Science, Tanta University, Tanta 31527, Egypt; 4 Department of Pharmaceutical Sciences, College of Pharmacy, AlMaarefa University, Riyadh 13713, Saudi Arabia; ikaeed@mcst.edu.sa; 5 Pharmaceutical Medicinal Chemistry & Drug Design Department, Faculty of Pharmacy (Boys), Al-Azhar University, Cairo 11884, Egypt

**Keywords:** 2′-dihalo ribonucleotides, HCV, molecular modeling and simulations, DFT studies

## Abstract

Sofosbuvir is the first approved direct-acting antiviral (DAA) agent that inhibits the HCV NS5B polymerase, resulting in chain termination. The molecular models of the 2′-dihalo ribonucleotides used were based on experimental biological studies of HCV polymerase inhibitors. They were modeled within HCV GT1a and GT1b to understand the structure–activity relationship (SAR) and the binding interaction of the halogen atoms at the active site of NS5B polymerase using different computational approaches. The outputs of the molecular docking studies indicated the correct binding mode of the tested compounds against the active sites in target receptors, exhibiting good binding free energies. Interestingly, the change in the substitution at the ribose sugar was found to produce a mild effect on the binding mode. In detail, increasing the hydrophobicity of the substituted moieties resulted in a better binding affinity. Furthermore, in silico ADMET investigation implied the general drug likeness of the examined derivatives. Specifically, good oral absorptions, no BBB penetration, and no CYP4502D6 inhibitions were expected. Likely, the in silico toxicity studies against several animal models showed no carcinogenicity and high predicted TD50 values. The DFT studies exhibited a bioisosteric effect between the substituents at the 2′-position and the possible steric clash between 2′-substituted nucleoside analogs and the active site in the target enzyme. Finally, compound 6 was subjected to several molecular dynamics (MD) simulations and MM-PBSA studies to examine the protein-ligand dynamic and energetic stability.

## 1. Introduction

Hepatitis C virus (HCV) is one of the major causes of chronic liver diseases and hepatocellular carcinoma (HCC) is the third leading cause of cancer death worldwide. The HCV nonstructural protein 5B (NS5B) is an RNA-dependent RNA polymerase (RdRp) that plays a vital role in RNA viral replication. Nucleoside/tide analogs have been used to treat viral infections and cancer for decades. Since the first FDA-approved nucleoside analog “edoxudine” in 1969, there are currently 25 FDA-approved nucleoside/tide analogs and another 15 nucleoside analogs used as antiviral and anticancer agents, respectively. Sofosbuvir (phosphoramidate prodrug of 2′-deoxy-2′-α-fluoro-β-C-methyluridine), a revolutionary HCV NS5B polymerase inhibitor and oral direct-acting antiviral (DAA), one of the best-selling drugs in the world, was approved by FDA in December 2013. Since its FDA approval, the cure rate has risen sharply to 90% after 12 weeks of treatment. Subsequently, the FDA approved many HCV drugs and currently play a pivotal role in HCV cure.

**Figure 1:**
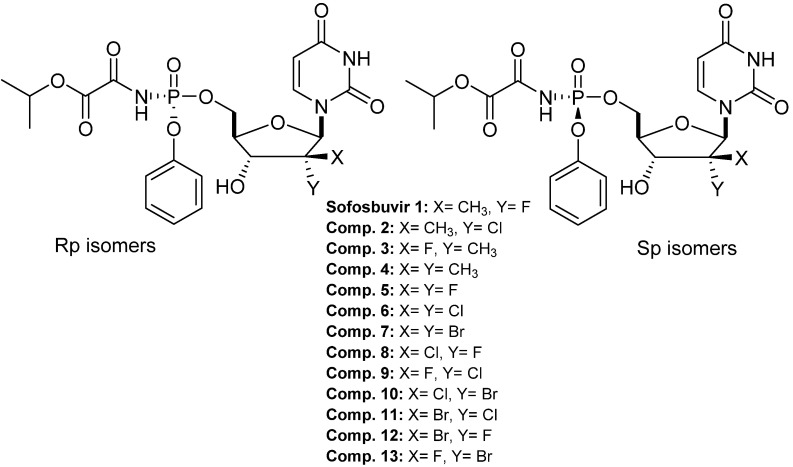
Sofosbuvir 1 (Sp isomer) and newly patented HCV prodrugs.

In April 2015, WHO added Daclatasvir (Daklinza™), Sofosbuvir (Sovaldi™), Ledipasvir (Harvoni™), Simeprevir (Olysio™), and Ombitasvir/Paritaprevir/r + Dasabuvir (Viekira Pak™) as essential WHO medicines based on the public health relevance and the medicines’ harms and benefits [1,2,3] (Figure 1).

Prodrugs are an inactive or less active form of pharmaceutical compounds, which are metabolized into an active form after administration to improve the delivery to the cell. Pronucleotides are prodrugs in which two different chemical linkers mask the monophosphate moiety, either oxygen to form phosphoester (phosphorous-oxygen bond) or nitrogen to form phosphoramidate (phosphorous–nitrogen bond, The ProTide Technology) prodrugs; phosphorous–nitrogen bonds in phosphoramidate prodrugs are intracellularly cleaved by phosphoramidase [4,5,6]. In 2013, the FDA approved the first direct-acting antiviral drug, Sovaldi^®^ (sofosbuvir), remarkably treating and curing HCV infection. Sofosbuvir is a phosphoramidate prodrug inhibitor of HCV polymerase.

**Figure 2:**
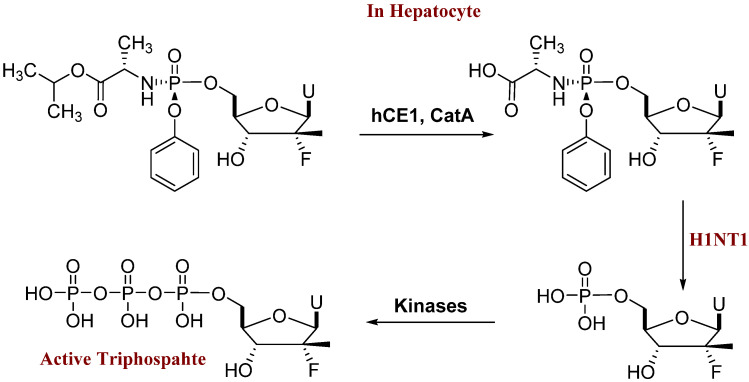
Sofosbuvir metabolism inside the hepatocyte. CatA = Cathepsin A; H1NT1 = histidine triad nucleotide-binding protein 1.

Sofosbuvir is the Sp isomer and has better activity than the Rp isomer [7]. Oral sofosbuvir is absorbed rapidly (*T_max_* ≈ 1 h), and all inactive metabolites are cleared mainly by the kidney. Sofosbuvir is phosphorylated to its active triphosphate in the hepatocytes, firstly by hydrolysis of the carbonyl ester in the phosphoramidate moiety by the hepatically expressed carbonyl esterase 1 (hCE1) and Cathepsin A (CatA). Further hydrolysis by histidine triad nucleotide-binding protein 1 (H1NT1) to its parent nucleoside (the monophosphate form) is further phosphorylated by phosphorylation kinases to nucleoside diphosphate and then to the triphosphate. This active form can compete with endogenous nucleotides, leading to the termination of HCV-RNA replication (Figure 2) [7,8,9].

Halogen atoms have a remarkable mimicking effect on many atoms and groups. The incorporation of one or more halogen atoms, especially the fluorine atom, into nucleoside analogs improved their pharmacological properties [10,11]. Recently, multiple research teams have discovered and developed novel 2′-deoxy-2′,2′-dihalonucleosides/tides with high potency against HCV NS5B polymerase [12,13,14,15,16,17,18]. Some of those analogs (e.g., compounds 6, 8, and 12) displayed better potency as compared to the marketed drug Sofosbuvir [12] due to the similarity of the van der Waal’s radius of the chlorine atom or bromine with the methyl group, which could provide the steric interference and the polarizability to terminate the RNA chain elongation of the viral replication (Table 1). This work aimed to study and understand the binding modes between the active 2′-deoxy-2′,2′-dihalonucleotides as a ligand, and NS5B polymerase as a receptor using modern computational techniques to realize the structural–activity relationship and to predict the ADMET/Tox properties of the active 2′-deoxy-2′,2′-dihalonucleotides compared with sofosbuvir.

## 2. Results and Discussion

### 2.1. Docking Studies

**Figure 3:**
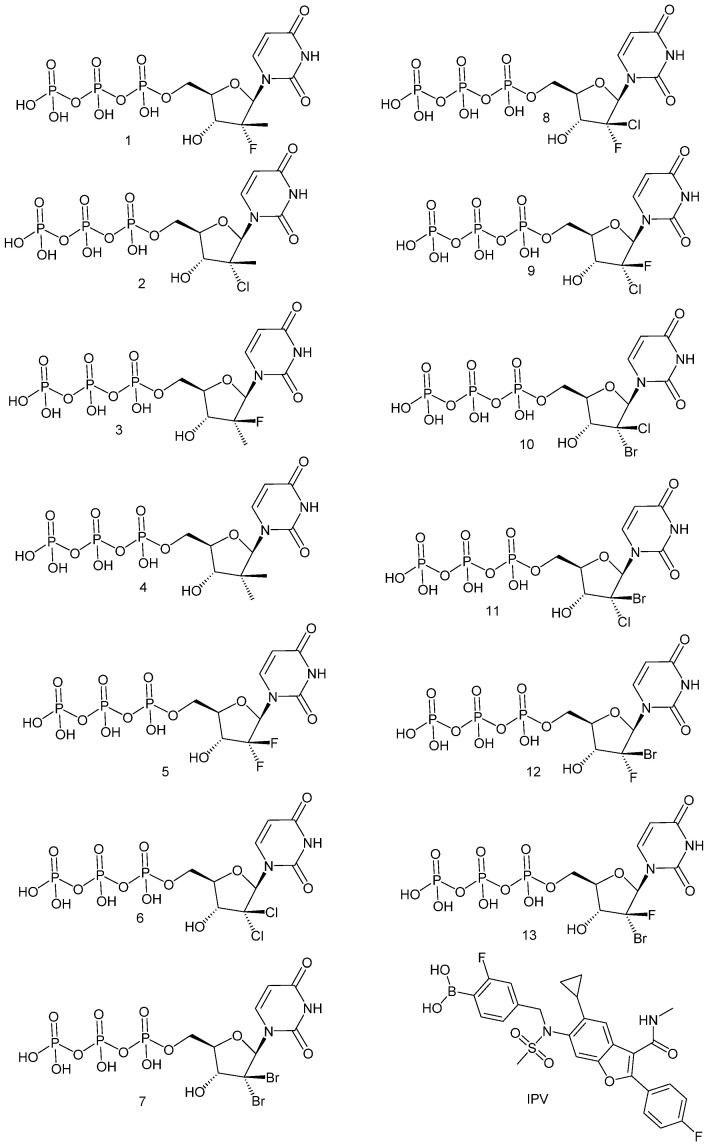
Structures of the docked compounds and the co-crystallized ligand.

Molecular docking studies were performed to obtain further insights into the binding modes and orientations of the examined compounds in the HCV RNA-dependent RNA polymerase or nonstructural protein 5B (NS5B), specifically the GT-1a and GT-1b subtypes (HCV NS5B GT1a and HCV NS5B GT1b) [19,20,21]. The target proteins were retrieved from Protein Data Bank (PDB ID for GT1a: 4khm and PDB ID for GT1b: 4kai). The co-crystalized ligand (IPV) was used as a reference ligand. This study evaluated how our small molecules (ligands) and the target macromolecules (HCV NS5B GT1a and HCV NS5B GT1b) fit together. Docking studies were carried out using MOE Software. The structures of the tested compounds and the co-crystallized ligand are presented in Figure 3. The docking scores are summarized in Table 2. From the results presented in Table 1, compounds **6** and **8** showed the highest activity against both HCV GT1a and GT1b. Accordingly, these compounds were selected for deep docking discussions.

#### 2.1.1. Docking Protocol Validation

**Figure 4:**
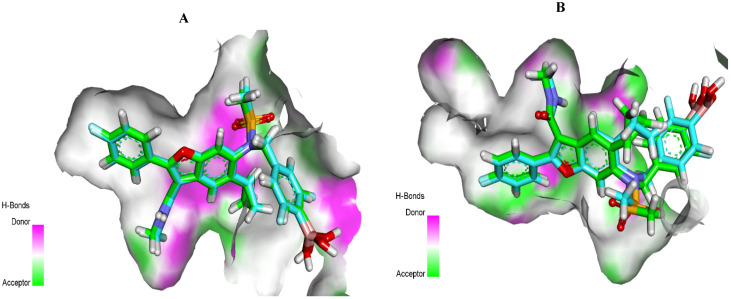
Superimposition of the co-crystallized pose (carbon atoms in turquoise) and the docking pose (carbon atoms in green) of the same ligand. (**A**) for GT1a, (**B**) for GTIb.

Validation of the docking protocol was achieved through re-docking of the co-crystallized ligands in the HCV NS5B GT1a and HCV NS5B GT1b active sites using MMFF94X as a force field and ASE as a scoring function. The validation step proved the suitability of the used protocol for the intended docking studies, as demonstrated by the small RMSD (1.12 and 1.23 Å for GT1a and GTIb, respectively) between the docked poses and the co-crystallized ligands. Moreover, the ability of the docking algorithm to retrieve the reported binding mode of the co-crystallized ligands also confirmed the validity of the selected docking algorithm (Figure 4).

#### 2.1.2. Docking Studies against GT1a

Firstly, the reported data indicated that six amino acids constitute the active site of HCV-NS5B GT1a, and they were involved in the interactions with HCV-NS5B inhibitors. These interacting residues are Arg200, Ser365, Gly192, Tyr316, Phe193, and Tyr448 [22].

**Figure 5:**
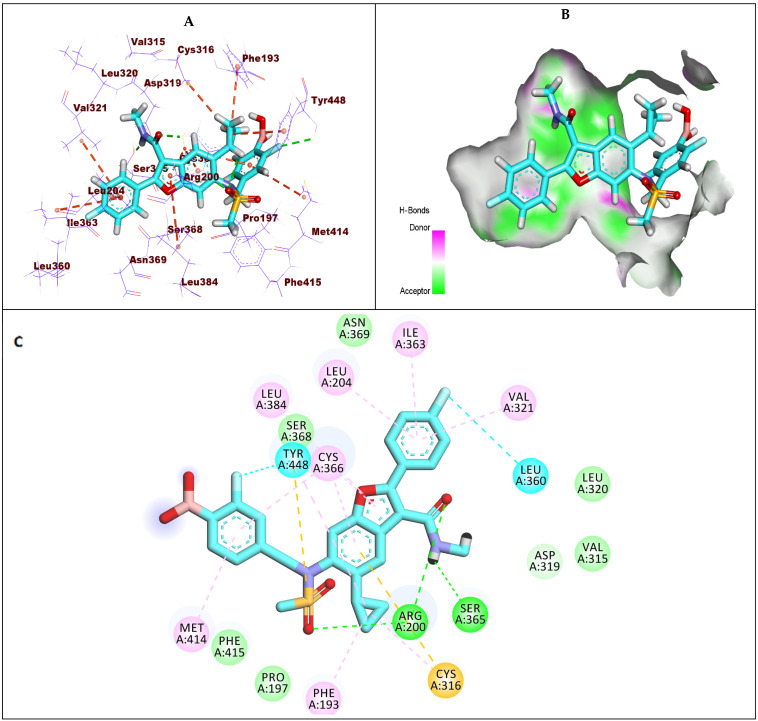
(**A**) The 3D structure of co-crystallized ligand, IPV, docked into the active site of HCV-NS5B GT1a; (**B**) mapping surface showing IPV occupying the active site of HCV-NS5B GT1a. (**C**) The 2D structure of co-crystallized ligand, IPV, docked into the active site of HCV-NS5B GT1a.

The co-crystalized ligand (IPV) displayed a −9.16 kcal/mol binding energy value against GT1a. The detailed binding mode was as follows: the (2-fluorophenyl)boronic acid moiety formed two hydrophobic interactions with Met414 and Cys366. Moreover, the fluoro atom formed a hydrogen bond with the amino acid Tyr448. Furthermore, the cyclohexyl moiety formed three hydrophobic interactions with Cys316, Phe193, and Tyr448. Additionally, the methylsulfonamide group formed two hydrogen bonds with Arg200. The N-methylbenzofuran-3-carboxamide moiety was incorporated into two hydrophobic interactions with Cys336 and Leu384. Moreover, it formed two hydrogen bonds with Arg200 and Ser365. Finally, the terminal 4-fluorophenyl moiety formed hydrophobic stacking with Ile363, Leu204, and Val321 (Figure 5).

**Figure 6:**
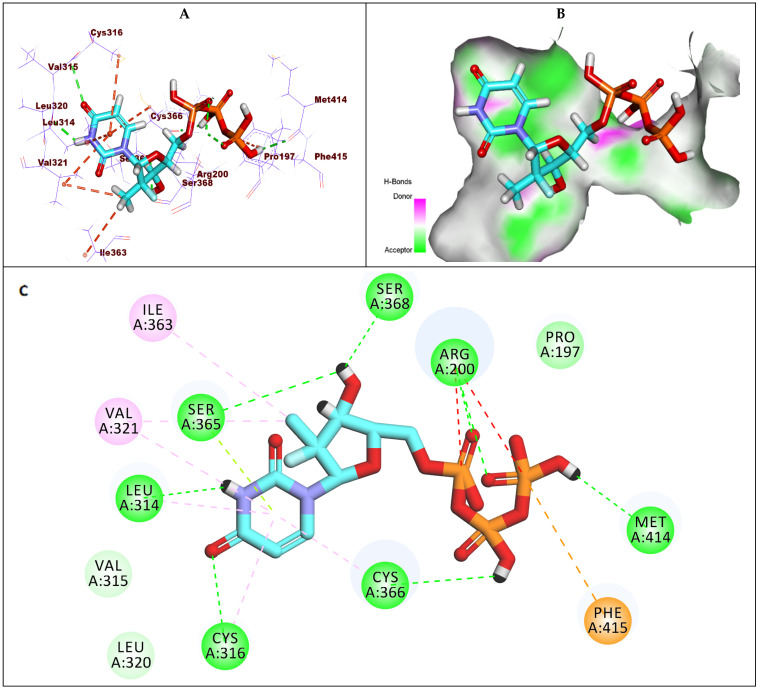
(**A**) The 3D structure of compound **1**, sofosbuvir, docked into the active site of HCV-NS5B GT1a; (**B**) mapping surface showing compound **1**, sofosbuvir, occupying the active site of HCV-NS5B GT1a; (**C**) The 2D structure of compound **1**, sofosbuvir, docked into the active site of HCV-NS5B GT1a.

Compound **1** (sofosbuvir) exhibited a −7.19 kcal/mol binding affinity value against GT1a. The triphosphate moiety formed five hydrogen bonds with Arg200, Cys336, and Met414. Additionally, it formed a hydrophobic interaction with Phe415. The hydroxyl group of the 2′-deoxy-2′-α-fluoro-2′-β-methylribose moiety formed two hydrogen bonds with the amino acids Ser368 and Ser365. Moreover, it formed two hydrophobic interactions with Ile363 and Val321. The uracil base formed two hydrogen bonds with Leu314 and Cys316. Furthermore, it formed four hydrophobic interactions with Cys366, Cys316, Val321, and Leu314 (Figure 6).

**Figure 7:**
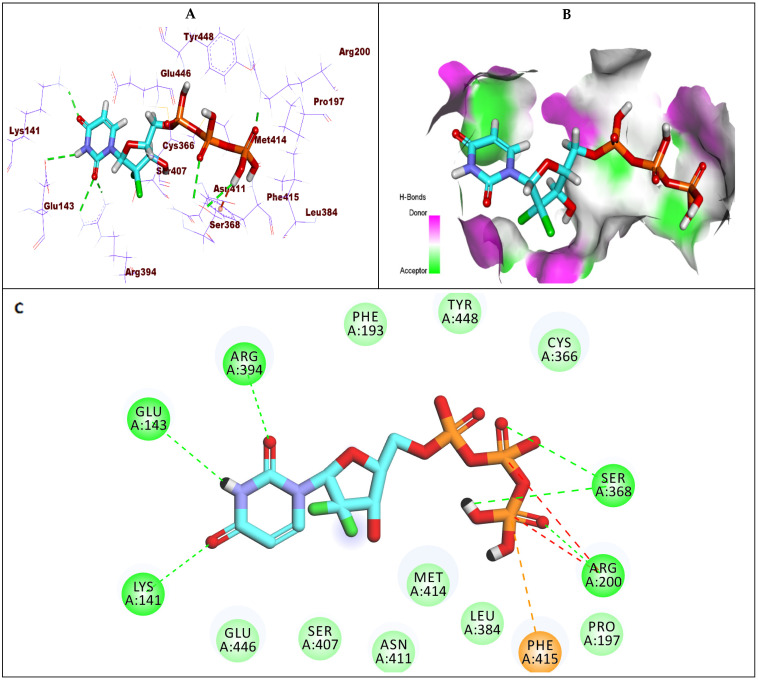
(**A**) The 3D structure of compound **6** docked into the active site of HCV-NS5B GT1a; (**B**) mapping surface showing compound **6** occupying the active site of HCV-NS5B GT1a; (**C**) The 2D structure of compound **6** docked into the active site of HCV-NS5B GT1a.

The binding mode of the active form of compound **6** is illustrated in Figure 7. It showed a binding energy of −6.21 kcal/mol against GT1a. The triphosphate moiety formed three hydrogen bonds with Ser368 and Arg200 in addition to electrostatic interaction with Phe415. The uracil base formed four hydrogen bonds with Arg394, Glu143, and Lys141.

**Figure 8:**
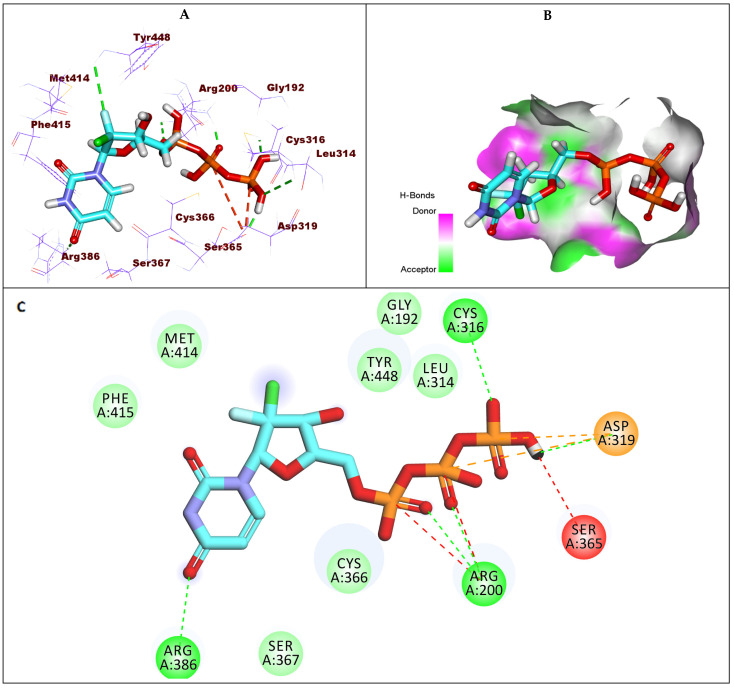
(**A**) The 3D structure of compound **8** docked into the active site of HCV-NS5B GT1a; (**B**) mapping surface showing compound **8** occupying the active site of HCV-NS5B GT1a; (**C**) The 2D structure of compound **8** docked into the active site of HCV-NS5B GT1a.

Compound **8** showed a binding energy of −6.79 kcal/mol against GT1a. The triphosphate moiety formed five hydrogen bonds with Asp319, Cys316, and Arg200 and two electrostatic interactions with Asp319. The fluoro atom of the 2′-deoxy-2′-β-chloro-2′-α-fluororibose moiety formed a hydrogen bond with Tyr448. Additionally, the uracil base formed another hydrogen bond with Arg386 (Figure 8).

#### 2.1.3. Docking Studies against GT1b

According to the previously reported data, the active site of HCV-NS5B **GT1b** involves six amino acids, including Arg200, Ser365, Gly192, Asn316, Phe193, and Tyr448. These amino acids were involved in interactions with HCV-NS5B inhibitors [22].

**Figure 9:**
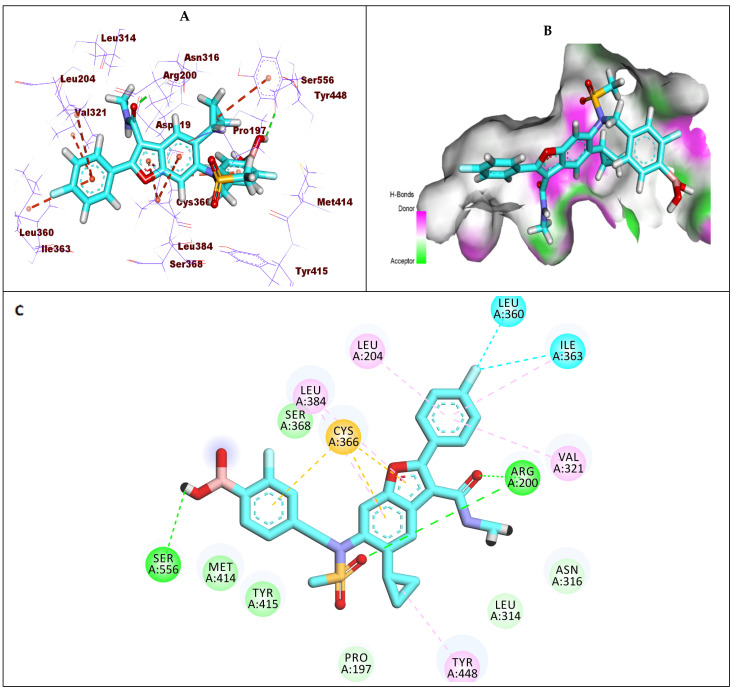
(**A**) The 3D structure of co-crystallized ligand, IPV, docked into the active site of HCV-NS5B GT1b; (**B**) mapping surface showing IPV occupying the active site of HCV-NS5B GT1b; (**C**) The 2D structure of co-crystallized ligand, IPV, docked into the active site of HCV-NS5B GT1b.

The co-crystalized ligand (IPV) showed a −7.24 kcal/mol binding energy value against GT1b. Each (2-fluorophenyl) boronic acid moiety and cyclohexyl moiety formed one hydrophobic interaction with Cys366 and Tyr448, respectively. The fluoro atom formed a hydrogen bond with Ser552. Additionally, the N-methyl benzofuran-3-carboxamide moiety was incorporated in four hydrophobic interactions with Cys366 and Leu384 in addition to forming a hydrogen bond with Arg200. Finally, the terminal 4-fluorophenyl moiety formed hydrophobic stacking with Ile363, Leu204, and Val321(Figure 9).

**Figure 10:**
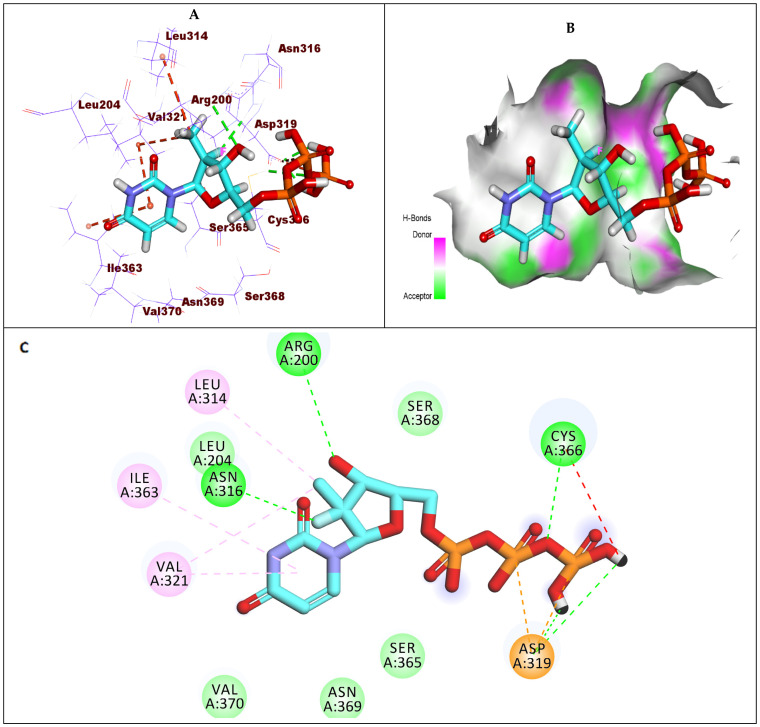
(**A**) The 3D structure of compound **1**, sofosbuvir, docked into the active site of HCV-NS5B GT1b; (**B**) mapping surface showing compound **1**, sofosbuvir, occupying the active site of HCV-NS5B GT1b; (**C**) The 2D structure of compound **1**, sofosbuvir, docked into the active site of HCV-NS5B GT1b.

Compound **1** (sofosbuvir) exhibited a −6.30 kcal/mol binding affinity value against GT1b. The triphosphate moiety formed three hydrogen bonds with Cys366 and Asp319. Additionally, it formed two hydrophobic interactions with Asp319. The hydroxyl group and fluoro atom of the 2′-deoxy-2′-α-fluoro-2′-β-methylribose moiety formed two hydrogen bonds with Asn316 and Arg200. In addition, it formed two hydrophobic interactions with Leu314 and Val321. The uracil base formed two hydrophobic interactions with Val321 and Ile363 (Figure 10).

**Figure 11:**
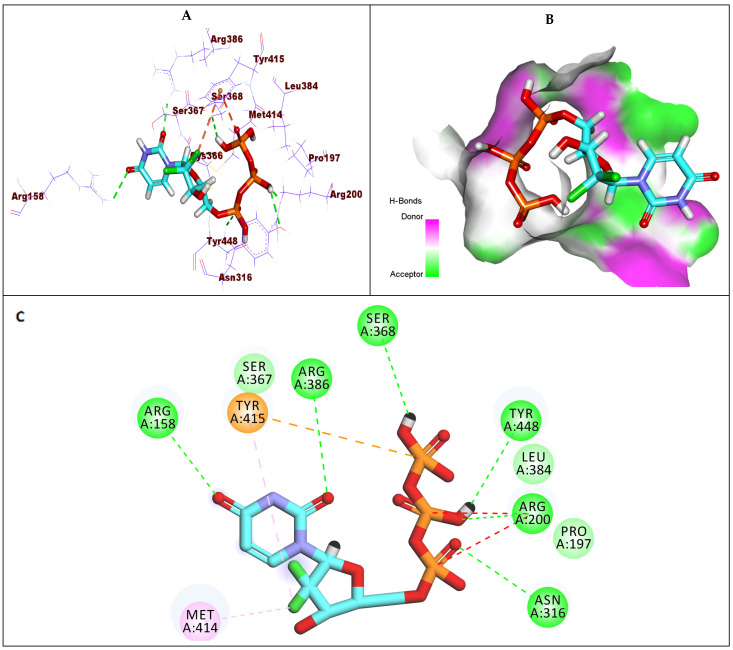
(**A**) The 3D structure of compound **6** docked into the active site of HCV-NS5B GT1b; (**B**) mapping surface showing compound **6** occupying the active site of HCV-NS5B GT1b; (**C**) The 2D structure of compound **6** docked into the active site of HCV-NS5B GT1b.

Compound **6** showed a binding energy of −6.63 kcal/mol against GT1b. The triphosphate moiety formed four hydrogen bonds with Arg200, Try448, Ser368, and Asn316, in addition to electrostatic interaction with Tyr415. The 2′-deoxy-2′,2′-dichlororibose moiety was incorporated in two hydrophobic interactions with Met414 and Tyr415. Finally, the uracil base formed two hydrogen bonds with Arg158 and Arg386 (Figure 11).

**Figure 12:**
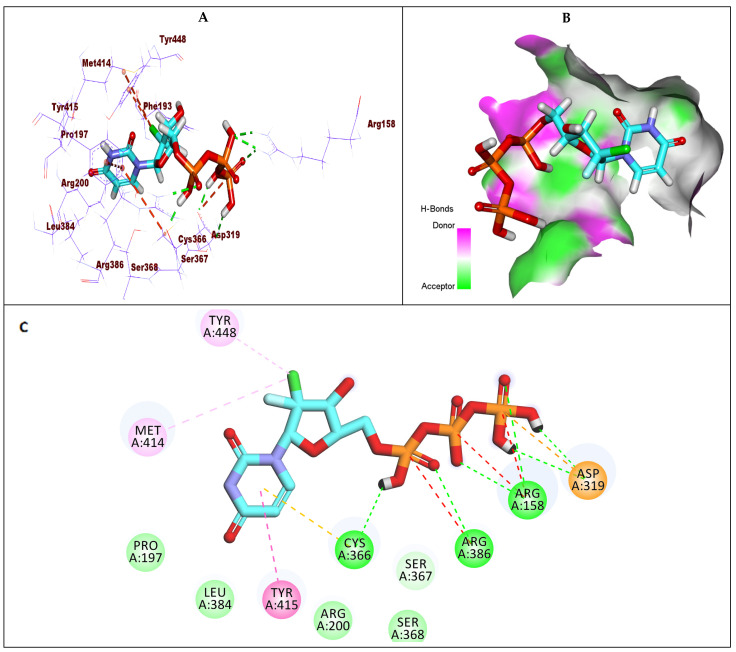
(**A**) The 3D structure of compound **8** docked into the active site of HCV-NS5B GT1b; (**B**) mapping surface showing compound **8** occupying the active site of HCV-NS5B GT1b; (**C**) The 2D structure of compound **8** docked into the active site of HCV-NS5B GT1b.

Compound **8** showed a binding energy of −6.39 kcal/mol against GT1b. The triphosphate moiety formed seven hydrogen bonds with Arg386, Arg158, Asp319, and Cys366, along with an electrostatic attraction with Asp319. The chloro atom of the 2′-deoxy-2′-β-chloro-2′-α-fluororibose moiety formed two hydrophobic interactions with Met414 and Tyr448. Furthermore, the uracil base formed two hydrophobic interactions with Cys366 and Tyr415 (Figure 12).

In conclusion, all examined compounds exhibited a good binding mode against the target receptors. It was noticed that the change in the substitution at the ribose sugar produced a mild effect on the binding potential. Specifically, it was found that the fluoro atom has more advantages than the other corresponding groups (chloro, bromo, and methyl). These advantages were confirmed by the increased biological activity of compounds substituted with a fluoro atom. In addition, as appeared in compound 8 (Figure 8), the fluoro atom has the ability to form a hydrogen bond at the active site, producing a significant fitting. Moreover, the chloro atom showed a good fitting with the receptors via its hydrophobic interactions as shown in compound 8 (Figure 12). From the binding mode of compound 1 (sofosbuvir) (Figure 6 and Figure 10), it is obvious that the methyl group has a significant role in the binding with the receptor via the formation of many hydrophobic interactions.

### 2.2. In Silico ADMET Analysis

In silico ADMET studies were carried out using sofosbuvir as a reference drug. The designed ADMET studies include the following descriptors: (i) blood–brain barrier penetration, which predicts the ability of a molecule to penetrate the blood–brain barrier; (ii) intestinal absorption, which predicts human intestinal absorption of a compound (HIA) after oral administration; (iii) aqueous solubility, which predicts the solubility of a compound in water at 25 °C; (iv) CYP2D6 binding predicts a molecule’s potential to inhibit the cytochrome P450 2D6 enzyme in the human body; and (v) plasma protein binding computes the fraction ratio of a molecule that is bound to plasma proteins in the bloodstream [23]. Discovery studio 4.0 software was utilized to investigate the ADMET descriptors for the presented compounds. The predicted ADMET descriptors are documented in Table 3.

The results revealed that all compounds exhibited a low or very low BBB penetration ability. Accordingly, such compounds were expected to be safe to CNS. All compounds were expected to have an optimal range of ADMET aqueous solubility levels. Intestinal absorption is the percentage of the absorbed compound from the human gut wall [24]. A well-absorbed compound is absorbed with a level of 90% or more into the bloodstream [25]. According to the in silico ADMET studies, all compounds were predicted to have good intestinal absorption levels.

**Figure 13:**
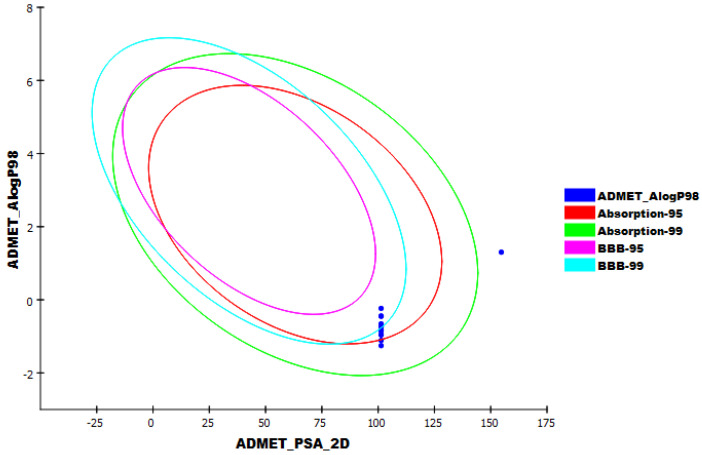
ADMET_AlogP98: lipid-water partition coefficient; ADMET_PSA_2D: polar molecular surface area. Each drug is plotted with a 2D polar surface area (PSA_2D) against its computed atom-type partition coefficient (ALogP98). The area encompassed by the ellipse represents good absorption without any violation of the ADMET properties. The 95% and 99% confidence limit ellipses corresponding to the blood–brain barrier (BBB) and intestinal absorption models are indicated.

The cytochrome P450 2D6 (CYP2D6) model computes the potential of a drug to inhibit the CYP2D6 enzyme by utilizing the 2D chemical structure of that drug as input. CYP2D6 is a vital enzyme responsible for the metabolism of a vast range of compounds inside the liver. Accordingly, its inhibition will cause a serious drug–drug interaction. Hence, the expectation of the CYP2D6 inhibition potential is demanded in the field of drug discovery and development [26]. All compounds were anticipated to be non-inhibitors of CYP2D6. Hence, no liver dysfunction side effect is predicted after its administration. The plasma protein-binding model calculates whether a molecule will bind highly (>=90%) to carrier plasma proteins in the blood or not [27]. The output of this experiment could classify whether a molecule is highly bound (>=90% bound) or not to plasma proteins by utilizing the Bayesian cutoff score of −2.209. All compounds were expected to bind plasma proteins with a level of less than 90% (Figure 13).

### 2.3. Toxicity Studies

Toxicity prediction was carried out based on the validated and constructed models in Discovery studio software [28,29] as follows: (i) FDA rodent carcinogenicity, which computes the probability of a submitted chemical structure being a carcinogen; (ii) carcinogenic potency TD_50,_ which computes the tumorigenic dose rate 50 (TD_50_) of a compound in a chronic exposure toxicity test for a rodent [30]; (iii) rat maximum tolerated dose (MTD) of the examined compounds [31,32]; (iv) rat chronic LOAEL, which predicts the rat chronic lowest observed adverse effect level (LOAEL) of a compound [33,34]; (v) ocular irritancy, which predicts the incidence and severity of ocular irritation of a particular compound in the Draize test (Wilhelmus, 2001 #41); and (vi) skin irritancy, which predicts the incidence and severity of the skin irritation of a particular compound in a rabbit skin irritancy test [35].

As shown in Table 4, the tested compounds showed in silico low toxicity against the tested models. For the FDA rodent carcinogenicity model, all examined compounds were expected to be non-carcinogenic.

For the carcinogenic potency TD_50_ rat model, the compounds showed TD_50_ values ranging from 4.059 to 5.463 mg/kg body weight/day. Such values are almost equal to the reference drug, sofosbuvir (4.929 mg/kg body weight/day). Regarding the rat maximum tolerated dose model, the compounds showed a maximum tolerated dose with a range of 0.021 to 0.063 g/kg body weight, which is almost equal to that of sofosbuvir (0.028 g/kg body weight). For the rat chronic LOAEL model, the tested compounds showed LOAEL values ranging from 0.0011 to 0.0026 g/kg body weight. These values are almost equal to or higher than sofosbuvir (0.0011 g/kg body weight). Moreover, all compounds were predicted to have a mild and moderate irritant effect against the skin irritancy and ocular irritancy models, respectively.

### 2.4. DFT Studies

The calculated values of the global chemical reactivity parameters and the free energy and the dipole moment for the parent nucleosides of the active phosphoramidate prodrugs are shown in Table 5. Compound **4** was excluded from the DFT studies, which focused only on 2′-halogenated nucleosides. The chemical reactivity values vary with different halogen substituents and their stereochemistry. The electronic transition from the HOMO to LUMO represents the energy gap (E_gap_), which describes the molecular reactivity. The value of the energy gap between HOMO and LUMO is directly proportional to the measure of molecules’ excitability; the smaller Egap in a molecule, the easier its electronic excitement between HOMO and LUMO and vice versa [36].

Moreover, a lower E_gap_ value would exhibit the eventual charge transfer interaction, which is generally responsible for the molecular bioactivity. Compounds **9** and **13** represent the highest Egap value (5.61 eV). Conversely, a larger E_gap_ provides lower chemical reactivity. The LUMO energy describes the electron affinity (EA) as the amount of energy released when an electron is added to the molecule to form a negative ion. A higher EA of a molecule suggests an electron acceptor in a charge-transfer reaction while a molecule with a lower EA is more likely to be an electron donor. Thus, 3, with the lowest EA (1.48), has a higher electron donor capacity while 6, representing the highest EA (1.98), has a lower donor capacity. Inversely, the energy of the HOMO describes the ionization potential (IP). The electrophilicity (ω) is an index used to quantify the global electrophilic nature, meaning that 7, which has the lowest ω (3.48), is the most electrophile. This index also measures the stabilization in energy when the system acquires an additional electronic charge; the smaller the (ω) value, the stronger the molecule’s stability.

The steric clash between 2′-substituted halogenated nucleoside analogs plays a pivotal role in the binding interaction of the 2′-β substitution and the active site in the target enzyme [18,19]. In order to understand the relationship between the van der Waals radii and volumes of 2′-substituted halogenated nucleosides and their biological activities, the Mulliken charges and bond length were calculated using DFT at the B3LYP/6-311+g(d,p) level of theory in addition to the Bondi radii and their volumes (Table 6) [37,38]. The results clearly indicate the bioisosteric effect between the methyl, chloro, and bromo groups, leading to similar biological activity.

Several studies were conducted in order to correlate different biological activities with chemical reactivity parameters such as the hardness, softness, chemical potential, electronegativity, electrophilicity, and other electronic parameters [36]. Simple linear regression of the biologically active and inactive parent nucleosides of the phosphoramidate prodrugs vs. global reactivity parameters of HCV genotypes 1a and 1b is shown in Table 7, representing the Pearson correlation coefficient (r) and the coefficient of determination (r^2^). The significant statistical relationship (*p* < 0.05) of the Pearson coefficient between the global chemical reactivity parameters (descriptors) and the biological activity (predictors) against HCV genotype 1a using the B3LYP/6-311+g(d,p) level of theory was obtained for E_gap_, global electron affinity, chemical potential, electrophilicity, hardness, and softness with an insignificant *p*-value (>0.05) for HCV GT1a and GT1b. However, the results were slightly improved for HCV genotype 1a and 1b with the global ionization potential (r*_I_* = 0.67 and 0.72) and electronegativity (rχ = 0.63 and 0.63) with a significant *p*-value (<0.05) for HCV GT1a and GT1b. the Pearson coefficient was positive and directly proportional to the biological activity. Multiple linear regression for statistically significant global reactivity indexes of the ionization potential and electronegativity was achieved and improved the Pearson correlation coefficient: r = 0.72 and r = 0.75 for GT1a and GT1b, respectively.

**Figure 14:**
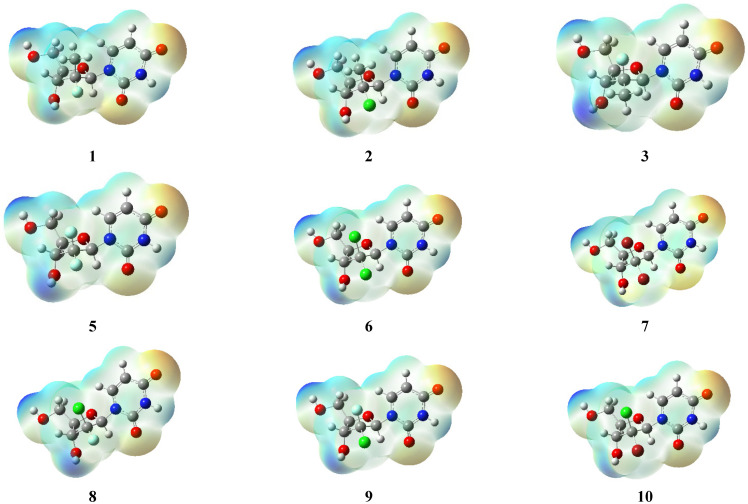

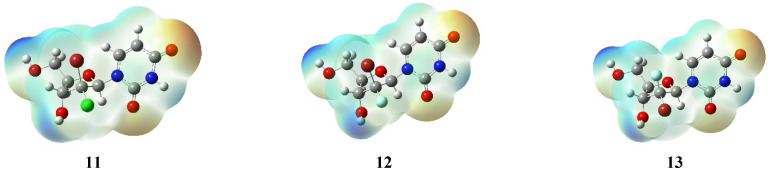
Optimized and calculated MEP maps for the halogenated nucleosides.

The parent 12 halogenated nucleosides were geometrically optimized at the ground-state using the density functional theory (DFT) at the B3LYP/6-311+g(d,p) level of theory, and MEP maps were calculated (Figure 14). The molecular electrostatic potential (MEP) is the potential that a positively charged unit would experience at any point surrounding the molecule due to the electron density distribution. The electrostatic potential may predict the chemical reactivity of molecules since regions of negative potential are expected to be sites of protonation and nucleophilic attack while regions of positive potential may indicate electrophilic sites.

### 2.5. Molecular Dynamics Simulation Studies

The molecular dynamics (MD) simulation experiment has an extra advantage over molecular docking, in addition to its ability to predict the binding mode correctly. It can predict the conformational changes of the ligand and the protein after the binding process [39]. Additionally, MD studies can accurately compute several parameters related to the change in the energy of the protein–ligand complex for a previously determined time. Accordingly, it precisely characterizes the stability, binding mode, and flexibility of the active compound inside the target enzyme [40]. The first MD simulation study of an enzyme was published in the *Nature* journal in 1977 [41]. Luckily, due to the recent supercomputer advancements, particularly the modern graphics processing parts, MD simulations analysis has become much more approachable, powerful, and precise [42]. The molecular docking and molecular dynamics simulations were performed for quinolinone derivatives with NS5B as a useful tool in order to design potent anti-HCV molecules [43].

**Figure 15:**
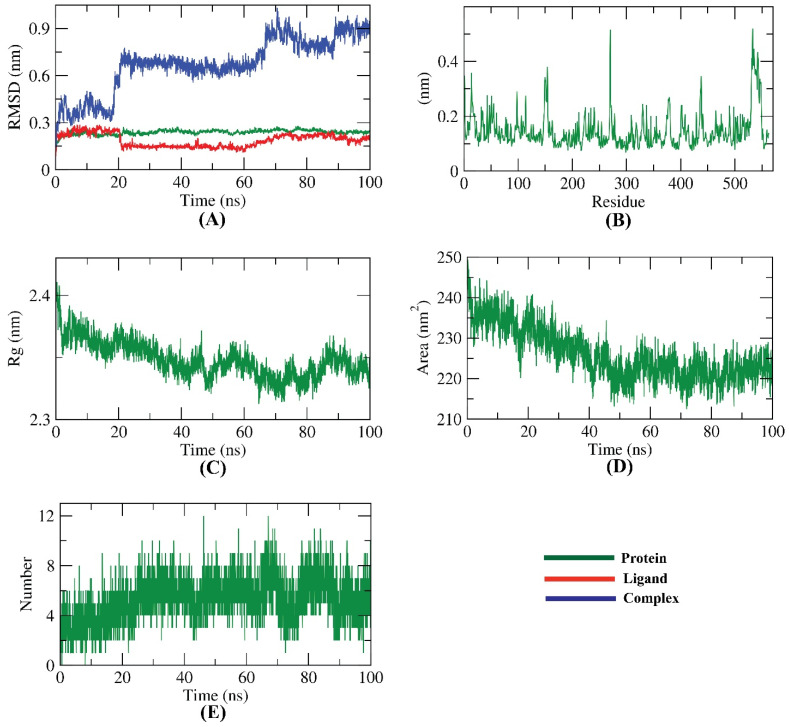
MD simulation results of HCV-NS5B GT1a–compound 6 complex; (**A**) RMSD, (**B**) RMSF, (**C**) Rg, (**D**) SASA, and (**E**) H- bonding.

Herein, the dynamics, together with the conformational variations of the HCV-NS5B GT1a-compound 6 complex, were calculated as a root mean square deviation (RMSD) over 100 ns. At first, the triphosphate groups were completely protonated, and Mg^++^ was removed. The results indicate that the HCV-NS5B GT1a, compound 6, and HCV-NS5B GT1a–compound 6 complex displayed lower RMSD values, indicating greater stability. Although the HCV-NS5B GT1a–compound 6 complex showed some fluctuations, it revealed good stability after 90 ns~ (Figure 15A). The flexibility of the HCV-NS5B GT1a residues was estimated in terms of the root mean square fluctuation (RMSF) to discover the fluctuated regions due to binding. As shown in Figure 15B, the binding of compound 6 did not cause major fluctuations in HCV-NS5B GT1a. Furthermore, the radius of gyration (Rg) was figured as a pivotal parameter associated with the protein constancy and stability [44,45]. The Rg of the HCV-NS5B GT1a–compound 6 complex (Figure 15C) was computed over 100 ns to be lower at the end of the study than the starting period. Additionally, the interaction between HCV-NS5B GT1a–compound 6 complex and the encircling solvents was estimated by the solvent accessible surface area (SASA) over 100 ns. SASA of the HCV-NS5B GT1a–compound 6 complex was calculated to explore the conformational changes that occurred due to binding. Interestingly, HCV-NS5B GT1a demonstrated a decrease in the SASA value through the 100 ns of the study (Figure 15D). The MD simulation studies revealed that the highest number of hydrogen bond conformations of the HCV-NS5B GT1a–compound 6 complex that was formed was up to 10 hydrogen bonds (Figure 15E).

### 2.6. MM-PBSA Studies

**Figure 16:**
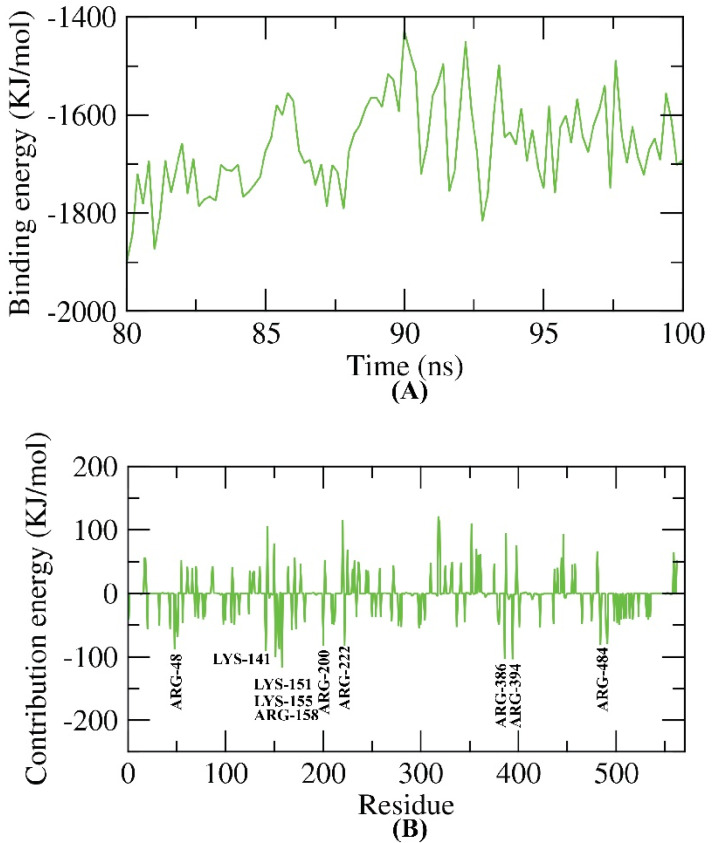
MM-PBSA results of the HCV-NS5B GT1a–compound 6 complex.

The MM-PBSA is an advanced, reliable tool used to estimate the free binding energy of a compound and protein. MM-PBSA is favored over other approaches such as the free energy perturbation and the thermodynamic integration because it is simpler, faster, and produces more consistent results [46]. The binding free energy of the HCV-NS5B GT1a–compound 6 complex was estimated in the final 20 ns of the MD study at an interval of 100 ps from the MD trajectories. Compound 6 demonstrated a low binding free energy of −1663 KJ/mol with HCV-NS5B GT1a (Figure 16A). Furthermore, the total binding energy of the HCV-NS5B GT1a–compound 6 complex was fragmented to discover the share of every amino acid residue of the HCV-NS5B GT1a in the binding with compound 6.

By breaking down the total binding energy of the HCV-NS5B GT1a–compound 6 complex into per amino acid residue share energy, this experiment discloses the ‘pivotal’ amino acid residues that contributed effectively to the binding of compound 6 to HCV-NS5B GT1a. It was found that the ARG-48, LYS-141, LYS-151, LYS-155, ARG-158, ARG-200, ARG-222, ARG-386, ARG-394, and ARG-484 residues of HCV-NS5B GT1a contributed more than a −80 KJ/mol binding energy (Figure 16B).

## 3. Experimental

### 3.1. Docking Studies

The crystal structures of the target proteins: (i) non-structural protein 5B (NS5BGT-1a) (PDB ID: 4khm, resolution: 1.70 Å) and (ii) non-structural protein 5B (NS5BGT-1b) (PDB ID: 4kai, resolution: 2.30 Å), were obtained from Protein Data Bank (http://www.pdb.org, accessed on 18 November 2020). Molecular Operating Environment (MOE) was utilized for the docking analysis [47]. The free binding energies and the binding modes of the tested compounds against the protein 5B were discussed. Firstly, the water molecules were removed from protein 5B’s crystal structure, leaving one chain, which is vital for the binding. IPV, the co-crystallized ligand, was utilized as a reference ligand. Next, protein 5B’s structure was protonated, and the hydrogen atoms were hidden. Additionally, all the triphosphate groups were completely protonated without any charge. Then, the binding pockets of the protein 5B were defined, and the energy was minimized [47,48].

The structures of the tested molecules and IPV (Figure 10) were drawn using ChemBioDraw Ultra 14.0 and kept as SDF files. Then, they were opened utilizing MOE software, and the 3D structures were protonated. Next, the compound’s energy was minimized. Validation processes for the target protein were carried out by the re-docking process for IPV. The performance validity depends on the low RMSD values between the docked and crystal conformations. Validation of the docking protocol was achieved through re-docking of the co-crystallized ligands in the HCV NS5B GT1a and HCV NS5B GT1b active sites using MMFF94X as a force field and ASE as a scoring function. The docking procedures were carried out through the default protocol. A set of 30 docked structures were generated utilizing the genetic algorithm searches. The outputted results from MOE software were visualized on Discovery Studio 4.0 software.

### 3.2. ADMET

The ADMET usual descriptors (absorption, distribution, metabolism, excretion, and toxicity) of the examined molecules were investigated using Discovery studio 4.0. At first, the CHARMM force field was applied and then the examined molecules were prepared and minimized according to the protocol of small molecule preparation. Finally, the ADMET descriptors protocol was applied.

### 3.3. Toxicity

The 2D structures of the examined molecules were drawn using ChemBioDraw Ultra 14.0 and saved as MDL-SD files. The 3D structures were protonated, and the energy was minimized using the MMFF94 force field for the partial charge and CHARMM force fields for the charge. Next, the examined molecules were prepared through the prepared ligand option. Additionally, the generation of tautomers, isomers, and fixation of bad valences was adjusted as false. Then, the toxicity of the prepared molecules was calculated by the option of toxicity prediction (extensible). Additionally, the similarity search was adjusted as true [49].

### 3.4. DFT Studies

DFT calculations were performed for the parent nucleosides using Gaussian 09 at the B3LYP/6-311+g(d,p) level of theory [50]. The parent nucleosides were geometrically optimized at the ground state using the density functional theory (DFT) at the B3LYP/6-311+g(d,p) level of theory in the gas phase and visualized using GaussView 6.0. The cartesian coordinates (in A°) of the optimized structures for Compound 1, 2, 3, 5, 6, 7, 8, 9, 10, 11, 12, 13 using B3LYP/6-311+g(d,p) level of theory are available in the Supplementary Materials. The global reactivity indexes for the investigated nucleosides were calculated using the Koopmans theorem: Ionization energy (I) = −E*_HOMO_*, electron affinity (EA) = −E*_LUMO_*, chemical potential (μ) = ½(I + A); electronegativity (χ) = −μ, hardness (η) = (I − A), softness (S) = 1/η, electrophilicity (ω) = χ^2^/2η.

### 3.5. MD Simulations

Among the tested compounds, the most promising compound **6** was advanced to MD simulations to study the relative stability of the protein–ligand interactions. The simulations were performed using the NAMD 2.13 package and the CHARMM36 force field. The parameters for the top docking results were generated using the CHARMM general force field (CgenFF). The TIP3P explicit solvation model was used, and the periodic boundary conditions were set with a dimension of the dimensions 95.56, 95.63, and 95.55 Å in x, y, and z, respectively. Afterward, the system was neutralized using 4 (Na+) ions. The MD protocols involved minimization, annealing, equilibration, and production. A 2-fs time step of integration was chosen for all MD simulations and the equilibration was carried out in the canonical (NVT) ensemble while the isothermal–isobaric (NPT) ensemble was for the production. Through the 100 ns of MD production, the pressure was set at 1 atm using the Nose’–Hoover Langevin piston barostat with a Langevin piston decay of 0.05 ps and a period of 0.1 ps. The temperature was set at 298.15 K using the Langevin thermostat. A distance cutoff of 12.0 Å was applied to short-range nonbonded interactions with a pair list distance of 16 Å, and Lennard-Jones interactions were smoothly truncated at 8.0 Å. Long-range electrostatic interactions were treated using the particle-mesh Ewald (PME) method, where a grid spacing of 1.0 Å was used for all simulation cells. All covalent bonds involving hydrogen atoms were constrained using the SHAKE algorithm for consistency, and the same protocol for all MD simulations was applied.

## 4. Conclusions

Twelve 2′-halogenated nucleotide analogs with a potential HCV polymerase inhibitory effect were subjected to many computational techniques, including docking, ADMET, toxicity, QSAR, DFT, and molecular dynamics simulation studies. Docking studies were performed against HCV GT1a and GT1b using sofosbuvir as a reference compound. The tested compounds exhibited a good binding mode against the target receptors, with a mild effect for the substitution of the ribose sugar on the binding potential. In addition, it was found that the high hydrophobic group can produce a higher binding affinity. The ADMET studies revealed that the tested compounds exhibited a low BBB penetration level, an optimal range of aqueous solubility, good intestinal absorption levels, a non-inhibitory effect on CYP2D6, and a plasma protein-binding ability of less than 90%. Moreover, the toxicity studies exhibited that the tested compounds were expected to be non-carcinogenic and equal to sofosbuvir regarding the carcinogenic potency TD_50_, maximum tolerated dose, and LOAEL values. Compound 6 was further subjected to molecular dynamics (MD) simulation to explain the protein–ligand complex stability and contacts. With these encouraging results, all the compounds can be further explored for structural modification and detailed investigations to arrive at possibly newer potent anti-HCV agents with better therapeutic activity.

## Figures and Tables

**Table 1:** HCV GT1a and GT1b replicon activity and cytotoxicity for the prodrugs 1–13 [12]. copyright has been obtained from Elsevier BMC, License Number 5346420180398, and it will be provided.

Compound	X	Y	EC_50_ GT1a	EC_50_ GT1b	CC_50_
**1**; Sofosbuvir	CH_3_	F	0.155	0.230	>100
**2**	CH_3_	Cl	0.139	0.167	>100
**3**	F	CH_3_	>10	>10	>32
**4**	CH_3_	CH_3_	5.63	4.75	31.6
**5**	F	F	0.226	0.286	0.384
**6**	Cl	Cl	0.043	0.048	>100
**7**	Br	Br	0.408	0.522	>100
**8**	Cl	F	0.053	0.061	11
**9**	F	Cl	0.9	NR	1.3
**10**	Cl	Br	0.114	0.147	80
**11**	Br	Cl	0.200	0.258	>100
**12**	Br	F	0.056	0.087	>100
**13**	F	Br	3.03	2.50	26.5

**Table 2:** The docking binding free energies (kcal/mol) of the title compounds and the co-crystallized ligand against GTIa and GTIb.

Comp.	1	2	3	4	5	6	7	8	9	10	11	12	13	Ligand
GTIa	−7.19	−6.65	−6.44	−6.73	−6.53	−6.21	−6.87	−6.79	−6.74	−7.27	−6.48	−7.21	−7.57	−9.16
GTIb	−6.30	−6.87	−6.79	−6.95	−6.45	−6.63	−7.04	−6.39	−7.09	−7.34	−6.37	−6.57	−6.68	−7.24

**Table 3:** Predicted ADMET results.

Comp.	BBB Level ^a^	Solubility Level ^b^		Absorption Level ^c^	CYP2D6 Prediction ^d^		PPB Prediction ^e^	
**1** (Sofosbuvir)	**4**	**3**	−3.72	1	−7.28	FALSE	1.30	FALSE
**2**	4	4	−0.66	0	−3.34	FALSE	−0.85	FALSE
**3**	4	4	−0.45	1	−2.51	FALSE	−1.11	FALSE
**4**	4	4	−0.41	0	−3.19	FALSE	−0.83	FALSE
**5**	4	4	−0.60	1	−2.90	FALSE	−1.25	FALSE
**6**	3	4	−1.09	0	−3.09	FALSE	−0.66	FALSE
**7**	3	4	−1.45	0	−5.45	FALSE	−0.24	FALSE
**8**	4	4	−0.84	0	−2.66	FALSE	−0.96	FALSE
**9**	4	4	−0.84	0	−2.66	FALSE	−0.96	FALSE
**10**	3	4	−1.27	0	−5.49	FALSE	−0.45	FALSE
**11**	3	4	−1.27	0	−5.49	FALSE	−0.45	FALSE
**12**	3	4	−1.02	0	−2.65	FALSE	−0.75	FALSE
**13**	3	4	−1.02	0	−2.65	FALSE	−0.75	FALSE

^a^ BBB level, blood–brain barrier level, 0 = very high, 1 = high, 2 = medium, 3 = low, 4 = very low. ^b^ Solubility level, 1 = very low, 2 = low, 3 = good, 4 = optimal. ^c^ Absorption level, 0 = good, 1 = moderate, 2 = poor, 3 = very poor. ^d^ CYP2D6, cytochrome P2D6, TRUE = inhibitor, FALSE = non inhibitor. The classification of whether a compound is a CYP2D6 inhibitor using the cutoff Bayesian score of 0.161. ^e^ PBB, plasma protein binding, FALSE means less than 90%, TRUE means more than 90%.

**Table 4:** Toxicity properties of the tested compounds.

Comp.	FDA Rodent Carcinogenicity (Rat- Male)	Carcinogenic Potency TD_50_ Rat ^a^	Rat Maximum Tolerated Dose (Feed) ^b^	Rat Chronic LOAEL ^b^	Skin Irritancy	Ocular Irritancy
**1** (Sofosbuvir)	Non-Carcinogen	4.929	0.028	0.0011	None	Moderate
** 2 **	Non-Carcinogen	4.059	0.051	0.0013	Mild	Moderate
** 3 **	Non-Carcinogen	4.101	0.056	0.0013	Mild	Moderate
** 4 **	Non-Carcinogen	5.463	0.063	0.0026	Mild	Moderate
** 5 **	Non-Carcinogen	4.998	0.050	0.0011	Mild	Moderate
** 6 **	Non-Carcinogen	4.810	0.042	0.0011	Mild	Moderate
** 7 **	Non-Carcinogen	5.159	0.021	0.0013	Mild	Moderate
** 8 **	Non-Carcinogen	4.911	0.046	0.0011	Mild	Moderate
** 9 **	Non-Carcinogen	4.911	0.046	0.0011	Mild	Moderate
** 10 **	Non-Carcinogen	5.024	0.030	0.0012	Mild	Moderate
** 11 **	Non-Carcinogen	5.024	0.030	0.0012	Mild	Moderate
** 12 **	Non-Carcinogen	5.169	0.033	0.0012	Mild	Moderate
** 13 **	Non-Carcinogen	5.169	0.033	0.0012	Mild	Moderate

^a^ mg/kg body weight/day. ^b^ Unit: g/kg body weight.

**Table 5:** The free energy in kcal.mol−1, dipole moment (Debye), and global reactivity descriptors (eV) for the analyzed parent nucleosides using B3LYP/6-311+g(d,p).

B3LYP/6-311+g(d,p)													
Comp.	X	Y	G	μ (D)	ε_LUMO_	ε_HOMO_	Egap	IP	EA	χ	η	S	ω
**1**	CH_3_	F	−611,483.98	6.44	−1.64409	−7.14044	5.50	7.14	1.64	4.39	5.50	0.18	1.75
**2**	CH_3_	Cl	−837,608.55	6.47	−1.64082	−7.15921	5.52	7.16	1.64	4.40	5.52	0.18	1.75
**3**	F	CH_3_	−611,488.44	5.77	−1.47538	−7.03921	5.56	7.04	1.48	4.26	5.56	0.18	1.63
**5**	F	F	−649,124.45	5.83	−1.65171	−7.24656	5.59	7.25	1.65	4.45	5.59	0.18	1.77
**6**	Cl	Cl	−1,101,368.36	5.78	−1.60926	−7.20493	5.60	7.20	1.61	4.41	5.60	0.18	1.74
**7**	Br	Br	−37,54,376.37	5.68	−1.98205	−7.18942	5.21	7.19	1.98	4.59	5.21	0.19	2.02
**8**	Cl	F	−875,244.85	5.97	−1.61878	−7.19813	5.58	7.20	1.62	4.41	5.58	0.18	1.74
**9**	F	Cl	−875,246.68	5.66	−1.63973	−7.2452	5.61	7.25	1.64	4.44	5.61	0.18	1.76
**10**	Cl	Br	−2,427,872.55	5.62	−1.68926	−7.20493	5.52	7.20	1.69	4.45	5.52	0.18	1.79
**11**	Br	Cl	−2,427,872.17	5.82	−1.82695	−7.1867	5.36	7.19	1.83	4.51	5.36	0.19	1.89
**12**	Br	F	−2,201,748.52	5.99	−1.60871	−7.17935	5.57	7.18	1.61	4.39	5.57	0.18	1.73
**13**	F	Br	−2,201,750.54	5.52	−1.64518	−7.25146	5.61	7.25	1.65	4.45	5.61	0.18	1.76

**Table 6:** Mulliken charges’ distribution, van der Waals radii, and volumes of 2′-substituted nucleosides.

			Mulliken Charges			Bond Length (Å)		Bondi Radii of Atoms and Their Volumes			
								X		Y	
Comp.	X	Y	C-2′	X	Y	C-X	C-Y	*R*_Bondi_ (Å)	*V*_vdW_ (Å^3^)	*R*_vdW_ (Å)	*V*_vdW_ (Å^3^)
**1**	CH_3_	F	0.509	C (−0.413) H (0.193, 0.188, 0.154)	−0.092	1.515	1.407	C (1.70) H (1.20)	C (20.58) H (7.24)	1.47	13.31
**2**	CH_3_	Cl	1.466	C (−0.665) H (0.191, 0.187, 0.156)	−0.004	1.524	1.825	C (1.70) H (1.20)	C (20.58) H (7.24)	1.75	22.45
**3**	F	CH_3_	0.398	−0.108	C (−0.309) H (0.190, 0.175, 0.169)	1.421	1.510	1.47	13.31	C (1.70) H (1.20)	C (20.58) H (7.24)
** 5 **	F	F	0.387	−0.076	−0.074	1.375	1.360	1.47	13.31	1.47	13.31
** 6 **	Cl	Cl	0.982	−0.254	0.237	1.811	1.790	1.75	22.45	1.75	22.45
** 7 **	Br	Br	0.971	0.004	0.074	1.984	1.959	1.85	26.52	1.85	26.52
** 8 **	Cl	F	0.347	0.119	−0.019	1.803	1.367	1.75	22.45	1.47	13.31
** 9 **	F	Cl	0.468	−0.026	0.222	1.384	1.784	1.47	13.31	1.75	22.45
** 10 **	Cl	Br	1.497	−0.324	0.102	1.809	1.963	1.75	22.45	1.85	26.52
** 11 **	Br	Cl	1.406	−0.007	0.033	1.986	1.787	1.85	26.52	1.75	22.45
** 12 **	Br	F	0.612	0.068	−0.028	1.977	1.366	1.85	26.52	1.47	13.31
** 13 **	F	Br	0.621	−0.033	0.048	1.384	1.956	1.47	13.31	1.85	26.52

**Table 7:** Pearson coefficient for simple and multiple linear regression of GT1a and GT1a.

B3LYP/6-311+g(d,p)				
HCV GT	Significant Indices	Linear Pearson Coefficient		
		r	r^2^	*p*
**GT1a**	Ionization Potential (I)	0.67	0.44	<0.05
	Electronegativity (χ)	0.63	0.39	<0.05
**GT1b**	Ionization Potential (I)	0.72	0.52	<0.05
	Electronegativity (χ)	0.63	0.40	<0.05
	**Significant Indices**	**Multiple Pearson Coefficient**		
**GT1a**	I and χ	0.72	0.51	<0.05
**GT1b**	I and χ	0.75	0.57	<0.05

## Data Availability

The data presented are available in the manuscript.

## Supplementary Materials

The following supporting information can be downloaded at: https://www.mdpi.com/article/10.3390/molecules27144530/s1, Tables S1–S12: Cartesian coordinates (in A°) of the optimized structures for Compound 1, 2, 3, 5, 6, 7, 8, 9, 10, 11, 12, 13 using B3LYP/6-311+g(d,p) level of theory.
