# Supplementary material for: The Inhibitory Potential of 2′-dihalo Ribonucleotides against HCV: Molecular Docking, Molecular Simulations, MM-BPSA, and DFT Studies
Source: Molecules. 2022 Jul 15;27(14):4530. doi: 10.3390/molecules27144530 (PMC9323285; doi:10.3390/molecules27144530)
Supplement: Supplementary file 1 [file molecules-27-04530-s001.zip › molecules-1790215-supplementary.pdf]

Supplementary Materials

The Inhibitory Potential of 2′-dihalo Ribonucleotides Against HCV: Molecular Docking, Molecular simulations, MM-BPSA, and DFT Studies

The supporting on-line material contains:

Tables S1-S12: Cartesian coordinates (in A°) of the optimized structures for Compound 1, 2, 3, 5, 6, 7, 8, 9, 10, 11, 12, 13 using B3LYP/6-311+g(d,p) level of theory.

Table S1: Cartesian coordinates (in A°) of the optimized structure for Compound 1 using B3LYP/6-311+g(d,p) level of theory.

Center Number	Atomic Number	Atomic Type	Coordinates (Angstroms) X	Y	Z
1	6	0	-2.003936	-0.316649	2.192152
2	1	0	-2.578023	-0.814121	1.438597
3	6	0	-0.395169	-0.970119	2.286432
4	1	0	-0.382314	-1.670124	3.045366
5	8	0	-0.094023	-1.348784	1.029005
6	6	0	0.521815	-0.398220	0.253339
7	1	0	1.712757	-0.777096	0.230361
8	7	0	0.196461	-0.155945	-1.030152
9	6	0	-0.910717	0.662701	-1.285144
10	1	0	-1.431077	1.142630	-0.482248
11	6	0	-1.390945	0.850364	-2.580668
12	1	0	-2.223483	1.495802	-2.719332
13	6	0	-0.689643	0.219492	-3.602493
14	8	0	-1.051909	0.375443	-4.855296
15	7	0	0.392493	-0.595274	-3.377962
16	1	0	0.869104	-1.036760	-4.113268
17	6	0	0.756027	-0.767542	-2.065966
18	8	0	1.801575	-1.530425	-1.837691
19	6	0	0.410210	0.384913	2.595156

20	1	0	-0.067830	1.022085	3.242932
21	6	0	0.983600	0.873439	1.248466
22	8	0	1.713210	-0.036352	3.203444
23	1	0	2.273989	0.745667	3.344355
24	8	0	-2.522518	-0.642430	3.575053
25	1	0	-3.413015	-1.022763	3.507102
26	1	0	-1.981110	0.740544	2.028682
27	6	0	0.077351	2.113338	1.134606
28	1	0	0.175496	2.709180	2.017918
29	1	0	0.367497	2.690041	0.281301
30	1	0	-0.940606	1.802281	1.025488
31	9	0	2.285965	0.864583	0.893128

Table S2: Cartesian coordinates (in A°) of the optimized structure for Compound 2 using B3LYP/6-311+g(d,p) level of theory.

Center Number	Atomic Number	Atomic Type	Coordinates (Angstroms) X	Y	Z
1	6	0	-0.238719	-1.159193	-0.018301
2	1	0	-0.204462	-0.266779	-0.607643
3	6	0	0.350965	-2.330510	-0.825697
4	1	0	-0.239545	-2.477019	-1.705886
5	8	0	1.766492	-2.001042	-1.082456
6	6	0	2.493761	-3.164730	-0.621511
7	1	0	2.446579	-3.955773	-1.340486
8	7	0	3.908829	-2.874546	-0.348975
9	6	0	4.276920	-1.634278	-0.002024
10	1	0	3.543388	-0.859207	0.076046
11	6	0	5.577291	-1.364215	0.249224

12	1	0	5.881235	-0.376627	0.527051
13	6	0	6.519139	-2.389226	0.141912
14	8	0	7.735208	-2.160767	0.371157
15	7	0	6.117391	-3.614944	-0.206435
16	1	0	6.785295	-4.354964	-0.285649
17	6	0	4.820517	-3.855417	-0.450166
18	8	0	4.453762	-5.013823	-0.777518
19	6	0	0.386303	-3.656375	-0.041011
20	1	0	-0.467052	-3.811771	0.585517
21	6	0	1.699237	-3.541966	0.635989
22	8	0	0.478851	-4.757251	-0.948979
23	1	0	0.586097	-5.572514	-0.453555
24	8	0	-1.595718	-1.452098	0.324716
25	1	0	-1.963313	-0.721926	0.828027
26	1	0	0.332685	-1.018603	0.875361
27	6	0	1.731095	-2.431880	1.702897
28	1	0	1.075459	-2.691671	2.507601
29	1	0	2.728197	-2.324123	2.075826
30	1	0	1.411765	-1.508551	1.266557
31	17	0	2.229885	-5.026594	1.418224

Table S3: Cartesian coordinates (in A°) of the optimized structure for Compound 3 using B3LYP/6-311+g(d,p) level of theory.

Center Number	Atomic Number	Atomic Type	Coordinates (Angstroms) X	Y	Z
1	6	0	-1.230089	-0.168142	0.000000
2	1	0	-1.530287	-1.024158	-0.615875
3	6	0	-1.859333	1.085378	-0.591873
4	1	0	-1.443971	1.258160	-1.588129

5	8	0	-3.281295	0.853874	-0.695104
6	6	0	-3.955181	2.063696	-0.380887
7	1	0	-3.945586	2.764873	-1.216486
8	7	0	-5.354766	1.759389	-0.116164
9	6	0	-5.722770	0.616843	0.569683
10	1	0	-4.897369	-0.005812	0.879370
11	6	0	-7.003719	0.277767	0.809833
12	1	0	-7.257169	-0.625130	1.344570
13	6	0	-8.084514	1.119162	0.324117
14	8	0	-9.275447	0.935112	0.483484
15	7	0	-7.624959	2.232699	-0.406782
16	1	0	-8.336775	2.833812	-0.803997
17	6	0	-6.325105	2.597715	-0.701667
18	8	0	-6.049952	3.554444	-1.392361
19	6	0	-1.673903	2.393337	0.215239
20	1	0	-0.950974	2.272282	1.028300
21	6	0	-3.104029	2.649896	0.777728
22	8	0	-1.239282	3.371107	-0.707112
23	1	0	-1.297794	4.241047	-0.292908
24	8	0	0.178948	0.024331	-0.016216
25	1	0	0.612899	-0.781755	0.277355
26	1	0	-1.596372	-0.336374	1.021147
27	6	0	-3.386559	4.154700	0.943085
28	1	0	-2.580559	4.612283	1.477764
29	1	0	-3.477798	4.608581	-0.021575
30	1	0	-4.297623	4.288781	1.487956
31	9	0	-3.286539	2.068646	1.982443

Table S4: Cartesian coordinates (in A°) of the optimized structure for Compound 5 using B3LYP/6-311+g(d,p) level of theory.

Center Number	Atomic Number	Atomic Type	Coordinates (Angstroms) X	Y	Z
1	6	0	-0.177467	-0.213714	0.198927
2	1	0	-0.224787	-1.071895	-0.438400
3	6	0	-0.760726	1.006038	-0.538374
4	1	0	-0.174708	1.194954	-1.413471
5	8	0	-2.179183	0.706375	-0.802200
6	6	0	-2.888923	1.850932	-0.272655
7	1	0	-2.835322	2.680396	-0.946463
8	7	0	-4.306007	1.561267	-0.010225
9	6	0	-4.686380	0.307592	0.268603
10	1	0	-3.961144	-0.478509	0.299871
11	6	0	-5.988637	0.037968	0.510373
12	1	0	-6.302354	-0.960266	0.734002
13	6	0	-6.919531	1.077489	0.464827
14	8	0	-8.137120	0.849722	0.686568
15	7	0	-6.505670	2.316353	0.183637
16	1	0	-7.165674	3.066815	0.148963
17	6	0	-5.207206	2.556053	-0.052263
18	8	0	-4.828995	3.726917	-0.316090
19	6	0	-0.777749	2.286411	0.317706
20	1	0	0.082838	2.398167	0.943646
21	6	0	-2.086322	2.150262	1.002871
22	8	0	-0.867053	3.436156	-0.527877
23	1	0	-0.961126	4.224146	0.012329
24	8	0	1.184737	0.046583	0.547559
25	1	0	1.548327	-0.713782	1.007176
26	1	0	-0.744169	-0.397790	1.087671
27	9	0	-2.115482	1.120622	1.875507
28	9	0	-2.477107	3.255343	1.672635

Table S5: Cartesian coordinates (in A°) of the optimized structure for Compound 6 using B3LYP/6-311+g(d,p) level of theory.

Center Number	Atomic Number	Atomic Type	Coordinates (Angstroms) X	Y	Z
1	6	0	-0.331458	1.740216	0.249457
2	1	0	-0.377003	0.908996	-0.422777
3	6	0	-0.923899	2.986448	-0.434297
4	1	0	-0.342408	3.214602	-1.303041
5	8	0	-2.341874	2.690557	-0.704883
6	6	0	-3.055266	3.808404	-0.125530
7	1	0	-3.008618	4.665394	-0.764506
8	7	0	-4.469782	3.500663	0.130092
9	6	0	-4.842642	2.234515	0.358070
10	1	0	-4.113298	1.451609	0.353875
11	6	0	-6.142519	1.948257	0.593441
12	1	0	-6.450246	0.939968	0.776625
13	6	0	-7.078862	2.983874	0.594692
14	8	0	-8.294367	2.740713	0.811466
15	7	0	-6.672449	4.235497	0.363609
16	1	0	-7.336394	4.983278	0.362674
17	6	0	-5.376188	4.491593	0.132875
18	8	0	-5.005009	5.674369	-0.083547
19	6	0	-0.943952	4.230071	0.474283
20	1	0	-0.081410	4.320260	1.101009
21	6	0	-2.249023	4.058730	1.158231
22	8	0	-1.042533	5.413444	-0.322467
23	1	0	-1.138415	6.177824	0.250359
24	8	0	1.030810	1.992960	0.603357

25	1	0	1.400124	1.216088	1.029594
26	1	0	-0.893602	1.516432	1.131962
27	17	0	-2.762256	5.459245	2.092443
28	17	0	-2.275594	2.670134	2.239292

Table S6: Cartesian coordinates (in A°) of the optimized structure for Compound 7 using B3LYP/6-311+g(d,p) level of theory.

Center Number	Atomic Number	Atomic Type	Coordinates (Angstroms) X	Y	Z
1	6	0	-1.353982	-1.194690	0.000000
2	1	0	-1.399527	-2.025910	-0.672234
3	6	0	-1.946423	0.051541	-0.683755
4	1	0	-1.364932	0.279695	-1.552498
5	8	0	-3.364399	-0.244350	-0.954341
6	6	0	-4.077791	0.873497	-0.374987
7	1	0	-4.031142	1.730488	-1.013963
8	7	0	-5.492306	0.565756	-0.119366
9	6	0	-5.865166	-0.700392	0.108612
10	1	0	-5.135823	-1.483297	0.104419
11	6	0	-7.165043	-0.986650	0.343983
12	1	0	-7.472770	-1.994939	0.527170
13	6	0	-8.101386	0.048968	0.345235
14	8	0	-9.316891	-0.194194	0.562009
15	7	0	-7.694973	1.300591	0.114151
16	1	0	-8.358918	2.048372	0.113215
17	6	0	-6.398713	1.556687	-0.116583
18	8	0	-6.027533	2.739463	-0.333003
19	6	0	-1.966477	1.295164	0.224826
20	1	0	-1.103934	1.385353	0.851552

21	6	0	-3.271547	1.123824	0.908774
22	8	0	-2.065057	2.478537	-0.571924
23	1	0	-2.160939	3.242918	0.000902
24	8	0	0.008286	-0.941947	0.353900
25	1	0	0.377600	-1.718818	0.780136
26	1	0	-1.916127	-1.418474	0.882505
27	35	0	-3.300383	-0.383119	2.081970
28	35	0	-3.828522	2.643700	1.922606

Table S7: Cartesian coordinates (in A°) of the optimized structure for Compound 8 using B3LYP/6-311+g(d,p) level of theory.

Center Number	Atomic Number	Atomic Type	Coordinates (Angstroms) X	Y	Z
1	6	0	0.433628	-2.221239	0.000000
2	1	0	0.388084	-3.052459	-0.672234
3	6	0	-0.158812	-0.975007	-0.683755
4	1	0	0.422678	-0.746853	-1.552498
5	8	0	-1.576788	-1.270898	-0.954341
6	6	0	-2.290180	-0.153052	-0.374987
7	1	0	-2.243532	0.703939	-1.013963
8	7	0	-3.704695	-0.460792	-0.119366
9	6	0	-4.077555	-1.726940	0.108612
10	1	0	-3.348212	-2.509846	0.104419
11	6	0	-5.377433	-2.013198	0.343983
12	1	0	-5.685160	-3.021487	0.527170
13	6	0	-6.313776	-0.977581	0.345235
14	8	0	-7.529280	-1.220743	0.562008
15	7	0	-5.907363	0.274042	0.114151
16	1	0	-6.571307	1.021823	0.113216

17	6	0	-4.611102	0.530138	-0.116583
18	8	0	-4.239922	1.712914	-0.333003
19	6	0	-0.178866	0.268616	0.224826
20	1	0	0.683677	0.358804	0.851552
21	6	0	-1.483937	0.097275	0.908774
22	8	0	-0.277446	1.451989	-0.571924
23	1	0	-0.373328	2.216369	0.000902
24	8	0	1.795897	-1.968495	0.353900
25	1	0	2.165211	-2.745367	0.780137
26	1	0	-0.128516	-2.445023	0.882505
27	17	0	-1.510508	-1.291322	1.989834
28	9	0	-1.877610	1.171533	1.625356

Table S8: Cartesian coordinates (in A°) of the optimized structure for Compound 9 using B3LYP/6-311+g(d,p) level of theory.

Center Number	Atomic Number	Atomic Type	Coordinates (Angstroms) X	Y	Z
1	6	0	0.044248	-1.336283	0.000000
2	1	0	-0.001297	-2.167503	-0.672234
3	6	0	-0.548193	-0.090051	-0.683755
4	1	0	0.033298	0.138102	-1.552498
5	8	0	-1.966168	-0.385943	-0.954341
6	6	0	-2.679560	0.731904	-0.374987
7	1	0	-2.632912	1.588895	-1.013963
8	7	0	-4.094076	0.424163	-0.119366
9	6	0	-4.466936	-0.841985	0.108612
10	1	0	-3.737592	-1.624890	0.104419
11	6	0	-5.766813	-1.128243	0.343983
12	1	0	-6.074540	-2.136532	0.527170

13	6	0	-6.703156	-0.092625	0.345235
14	8	0	-7.918661	-0.335787	0.562008
15	7	0	-6.296743	1.158998	0.114151
16	1	0	-6.960688	1.906779	0.113215
17	6	0	-5.000482	1.415094	-0.116583
18	8	0	-4.629303	2.597870	-0.333003
19	6	0	-0.568246	1.153571	0.224826
20	1	0	0.294296	1.243760	0.851552
21	6	0	-1.873317	0.982231	0.908774
22	8	0	-0.666827	2.336944	-0.571924
23	1	0	-0.762709	3.101325	0.000902
24	8	0	1.406516	-1.083540	0.353900
25	1	0	1.775830	-1.860411	0.780136
26	1	0	-0.517896	-1.560067	0.882505
27	9	0	-1.893699	-0.082886	1.737996
28	17	0	-2.386550	2.382745	1.842985

Table S9: Cartesian coordinates (in A°) of the optimized structure for Compound 10 using B3LYP/6-311+g(d,p) level of theory.

Center Number	Atomic Number	Atomic Type	Coordinates (Angstroms) X	Y	Z
1	6	0	1.690266	-0.274336	0.000000
2	1	0	1.644721	-1.105556	-0.672234
3	6	0	1.097825	0.971895	-0.683755
4	1	0	1.679316	1.200049	-1.552498
5	8	0	-0.320151	0.676004	-0.954341
6	6	0	-1.033543	1.793851	-0.374987
7	1	0	-0.986895	2.650842	-1.013963
8	7	0	-2.448058	1.486110	-0.119366

9	6	0	-2.820918	0.219962	0.108612
10	1	0	-2.091575	-0.562943	0.104419
11	6	0	-4.120795	-0.066296	0.343984
12	1	0	-4.428522	-1.074585	0.527170
13	6	0	-5.057138	0.969322	0.345235
14	8	0	-6.272643	0.726160	0.562008
15	7	0	-4.650725	2.220945	0.114151
16	1	0	-5.314670	2.968726	0.113215
17	6	0	-3.354465	2.477041	-0.116583
18	8	0	-2.983285	3.659817	-0.333003
19	6	0	1.077771	2.215518	0.224826
20	1	0	1.940314	2.305707	0.851552
21	6	0	-0.227300	2.044178	0.908774
22	8	0	0.979191	3.398891	-0.571924
23	1	0	0.883309	4.163272	0.000902
24	8	0	3.052534	-0.021593	0.353900
25	1	0	3.421848	-0.798464	0.780136
26	1	0	1.128121	-0.498120	0.882505
27	17	0	-0.253871	0.655581	1.989834
28	35	0	-0.784274	3.564054	1.922606

Table S10: Cartesian coordinates (in A°) of the optimized structure for Compound 11 using B3LYP/6-311+g(d,p) level of theory.

Center Number	Atomic Number	Atomic Type	Coordinates (Angstroms) X	Y	Z
1	6	0	-0.858407	-0.522124	0.000000
2	1	0	-0.903952	-1.353344	-0.672234
3	6	0	-1.450848	0.724108	-0.683755
4	1	0	-0.869357	0.952262	-1.552498

5	8	0	-2.868823	0.428217	-0.954341
6	6	0	-3.582215	1.546063	-0.374987
7	1	0	-3.535567	2.403054	-1.013963
8	7	0	-4.996731	1.238323	-0.119366
9	6	0	-5.369591	-0.027825	0.108612
10	1	0	-4.640247	-0.810731	0.104419
11	6	0	-6.669468	-0.314083	0.343983
12	1	0	-6.977195	-1.322372	0.527170
13	6	0	-7.605811	0.721534	0.345235
14	8	0	-8.821316	0.478372	0.562009
15	7	0	-7.199398	1.973157	0.114151
16	1	0	-7.863343	2.720938	0.113215
17	6	0	-5.903137	2.229253	-0.116583
18	8	0	-5.531958	3.412029	-0.333003
19	6	0	-1.470901	1.967731	0.224826
20	1	0	-0.608359	2.057919	0.851552
21	6	0	-2.775972	1.796390	0.908774
22	8	0	-1.569482	3.151104	-0.571924
23	1	0	-1.665364	3.915484	0.000902
24	8	0	0.503861	-0.269380	0.353900
25	1	0	0.873175	-1.046252	0.780136
26	1	0	-1.420551	-0.745908	0.882505
27	35	0	-2.804808	0.289447	2.081970
28	17	0	-3.289205	3.196904	1.842985

Table S11: Cartesian coordinates (in A°) of the optimized structure for Compound 12 using B3LYP/6-311+g(d,p) level of theory.

Center	Atomic	Atomic	Coordinates (Angstroms)		
Number	Number	Type	X	Y	Z

1	6	0	-1.017699	-1.371681	0.000000
2	1	0	-1.063244	-2.202901	-0.672234
3	6	0	-1.610140	-0.125450	-0.683755
4	1	0	-1.028649	0.102704	-1.552498
5	8	0	-3.028115	-0.421341	-0.954340
6	6	0	-3.741507	0.696506	-0.374987
7	1	0	-3.694859	1.553497	-1.013963
8	7	0	-5.156023	0.388765	-0.119365
9	6	0	-5.528883	-0.877383	0.108613
10	1	0	-4.799539	-1.660288	0.104419
11	6	0	-6.828760	-1.163641	0.343983
12	1	0	-7.136488	-2.171930	0.527168
13	6	0	-7.765103	-0.128024	0.345234
14	8	0	-8.980608	-0.371185	0.562008
15	7	0	-7.358690	1.123600	0.114152
16	1	0	-8.022635	1.871381	0.113217
17	6	0	-6.062429	1.379696	-0.116582
18	8	0	-5.691250	2.562471	-0.333003
19	6	0	-1.630193	1.118173	0.224826
20	1	0	-0.767650	1.208362	0.851552
21	6	0	-2.935264	0.946833	0.908774
22	8	0	-1.728774	2.301546	-0.571924
23	1	0	-1.824656	3.065926	0.000901
24	8	0	0.344569	-1.118938	0.353900
25	1	0	0.713883	-1.895809	0.780136
26	1	0	-1.579843	-1.595465	0.882505
27	35	0	-2.964100	-0.560110	2.081971
28	9	0	-3.328937	2.021091	1.625356

Table S12: Cartesian coordinates (in A°) of the optimized structure for Compound 13 using B3LYP/6-311+g(d,p) level of theory.

Center Number	Atomic Number	Atomic Type	Coordinates (Angstroms) X	Y	Z
1	6	0	-1.176991	-0.876106	0.000000
2	1	0	-1.222536	-1.707326	-0.672234
3	6	0	-1.769432	0.370125	-0.683755
4	1	0	-1.187941	0.598279	-1.552498
5	8	0	-3.187408	0.074234	-0.954340
6	6	0	-3.900800	1.192081	-0.374987
7	1	0	-3.854151	2.049072	-1.013963
8	7	0	-5.315315	0.884340	-0.119365
9	6	0	-5.688175	-0.381807	0.108613
10	1	0	-4.958831	-1.164713	0.104419
11	6	0	-6.988052	-0.668066	0.343983
12	1	0	-7.295780	-1.676355	0.527168
13	6	0	-7.924395	0.367552	0.345234
14	8	0	-9.139900	0.124390	0.562008
15	7	0	-7.517982	1.619175	0.114152
16	1	0	-8.181927	2.366956	0.113217
17	6	0	-6.221721	1.875271	-0.116582
18	8	0	-5.850542	3.058047	-0.333003
19	6	0	-1.789485	1.613748	0.224826
20	1	0	-0.926943	1.703937	0.851552
21	6	0	-3.094556	1.442408	0.908774
22	8	0	-1.888066	2.797121	-0.571924
23	1	0	-1.983948	3.561502	0.000902
24	8	0	0.185277	-0.623362	0.353900
25	1	0	0.554591	-1.400234	0.780136

**26**	**1**	**0**	**-1.739135**	**-1.099890**	**0.882505**
**27**	**9**	**0**	**-3.114937**	**0.377291**	**1.737997**
**28**	**35**	**0**	**-3.651531**	**2.962285**	**1.922606**
